# Genomic Selection for Weaning Weight in Alpine Merino Sheep Based on GWAS Prior Marker Information

**DOI:** 10.3390/ani14131904

**Published:** 2024-06-27

**Authors:** Haifeng Wang, Chenglan Li, Jianye Li, Rui Zhang, Xuejiao An, Chao Yuan, Tingting Guo, Yaojing Yue

**Affiliations:** 1 Key Laboratory of Animal Genetics and Breeding on Tibetan Plateau, Ministry of Agriculture and Rural Affairs, Lanzhou Institute of Husbandry and Pharmaceutical Sciences, Chinese Academy of Agricultural Sciences, Lanzhou 730050, China; wanghaifeng822@163.com (H.W.); 82101215420@caas.cn (C.L.); lijianye@caas.cn (J.L.); zhangrui03@caas.cn (R.Z.); 18393811056@163.com (X.A.); yuanchao@caas.cn (C.Y.); 2 Sheep Breeding Engineering Technology Research Center of Chinese Academy of Agricultural Sciences, Lanzhou 730050, China

**Keywords:** genomic selection, GWAS, prior marker information, prediction accuracy

## Abstract

**Simple Summary:**

This study compared the accuracy of estimating the breeding value of weaning weight traits in Alpine Merino sheep using a combination of genome-wide association analysis and genome selection with the best linear unbiased prediction method. The test population was randomly divided into two groups. One group was used for discovering marker information through GWAS analysis and screening the most significant loci, and the other group used different prior marker information to estimate genetic parameters and compare the prediction accuracy of genomic breeding values. The study demonstrates that integrating the genome selection method with prior marker information from GWAS can enhance the prediction accuracy of genome breeding value. This research offers technical support for future breeding efforts of Alpine Merino sheep and the enhancement of genomic breeding value accuracy.

**Abstract:**

This study aims to compare the accuracy of genomic estimated breeding values (GEBV) estimated using a genomic best linear unbiased prediction (GBLUP) method and GEBV estimates incorporating prior marker information from a genome-wide association study (GWAS) for the weaning weight trait in highland Merino sheep. The objective is to provide theoretical and technical support for improving the accuracy of genomic selection. The study used a population of 1007 highland Merino ewes, with the weaning weight at 3 months as the target trait. The population was randomly divided into two groups. The first group was used for GWAS analysis to identify significant markers, and the top 5%, top 10%, top 15%, and top 20% markers were selected as prior marker information. The second group was used to estimate genetic parameters and compare the accuracy of GEBV predictions using different prior marker information. The accuracy was obtained using a five-fold cross-validation. Finally, both groups were subjected to cross-validation. The study’s findings revealed that the heritability of the weaning weight trait, as calculated using the GBLUP model, ranged from 0.122 to 0.394, with corresponding prediction accuracies falling between 0.075 and 0.228. By incorporating prior marker information from GWAS, the heritability was enhanced to a range of 0.125 to 0.407. The inclusion of the top 5% to top 20% significant SNPs from GWAS results as prior information into GS showed potential for improving the accuracy of predicting genomic breeding value.

## 1. Introduction

Body weight traits are important indicators of economic traits in sheep and play a significant role in animal husbandry. They serve as core indicators for assessing the meat potential of sheep and are closely tied to the health status of individual animals. Additionally, body weight traits are closely associated with yield levels and greatly influence the economic benefits of farmers [1]. A deep understanding and mastery of the genetic patterns related to weight traits can lead to breakthroughs in genetic breeding efforts, thus advancing the sustainable and healthy progression of the sheep industry. However, traditional genetic breeding methods have progressed slowly in the study of weight traits, failing to meet the demands of modern animal husbandry development. The use of modern breeding technology offers a novel approach and methodology for developing high-quality sheep breeds at the molecular level. For instance, the application of modern breeding technology not only expedites the breeding process of Alpine Merino sheep but also enhances breeding accuracy and efficiency. This enables farmers to swiftly identify individuals with exceptional body weight traits and further develop high-quality sheep breeds that align better with market demands. By combining modern breeding techniques with traditional genetic breeding methods, we can gain a deeper understanding of and effectively utilize the genetic mechanisms related to weight traits, ultimately contributing to the sustainable advancement of animal husbandry.

The development and application of molecular markers, such as single-nucleotide polymorphisms (SNPs), have enabled the integration of functionally validated genetic markers with the genomic best linear unbiased prediction (GBLUP) method for molecular marker-assisted selection of breeding values. This not only enhances the accuracy of selection but also allows for early selection, effectively reducing the breeding time interval [2]. However, the majority of economic traits in poultry are quantitative traits governed by multiple minor-effect genes, making it challenging to identify loci with significant effects [3]. Genomic selection (GS), a high-density marker technique spanning the entire genome, was initially introduced by Meuwissen in 2001 [4]. Particularly beneficial for traits with low heritability and those difficult to measure, GS offers advantages such as shortened generation intervals, decreased breeding expenses [5,6,7], and enhanced precision in genomic estimated breeding value (GEBV) [8]. The GS approach encompasses direct methods like GBLUP and indirect methods like Bayes techniques. Though the latter boasts high accuracy in calculations, it is time-consuming and demands considerable computational resources, limiting its practical application [9]. In recent years, the reduced costs of high-throughput sequencing have led to the widespread adoption of GBLUP in livestock and poultry breeding, including pigs [10,11], cattle [12], sheep [13], and chickens [14], due to its efficient computation and user-friendly nature. However, the assumption in the GBLUP model that all SNPs have an equal and effective impact on traits is considered unrealistic. The genetic basis and complexity of various quantitative traits vary, leading to different genetic markers having differing effects. As a result, there remains potential for enhancement in the GBLUP methodology [15,16]. Previous studies have demonstrated that incorporating data from genome-wide association studies (GWAS) can enhance the accuracy of GS for important economic traits in livestock and poultry [17,18]. GWAS play a crucial role in identifying candidate loci associated with economic traits in poultry [19,20]. Various models, such as the General Linear Model (GLM), Mixed Linear Model (MLM), and FarmCPU model, are available for GWAS analysis. In this study, the FarmCPU model within the rMVP software package (https://github.com/xiaolei-lab/rMVP (accessed on 15 May 2023)) was employed for analysis. By combining the strengths of MLM and GLM, this model addresses the limitations between the genetic relationship and population structure relationship through iteration. This approach helps to distinguish genetic trait genes from target trait genes in selection, leading to a more effective reduction in errors.

The Alpine Merino sheep is a newly developed breed that has been successfully bred over a period of 20 years using advanced modern breeding techniques. It was created by crossing Australian Merino sheep as the male parent with Gansu Alpine Merino sheep as the female parent. The breeding process involved three main stages: hybridization improvement, cross fixation, and breeding improvement. The Alpine Merino sheep typically inhabits the highland grassland area of the Qilian Mountains, at altitudes ranging from 2400 to 4070 m throughout the year. Known for its large body size, abundant wool production, soft and delicate wool texture, good elasticity and warmth, and fresh and flavorful meat, the Alpine Merino sheep is recognized for its excellent quality. The breeding of this new breed not only enhanced the genetic resources of Chinese native sheep breeds but also played a vital role in boosting local economic growth in cold and arid mountainous regions. In sheep breeding, body weight serves as a crucial indicator of growth and development, impacting both wool quality and meat production. The early growth rate of sheep holds significant economic value. Though the use of GWAS and GS in animal breeding is well established, only a limited number of studies have utilized GWAS analysis results as prior information for GS prediction accuracy. This study aimed to compare the accuracy of breeding value estimation for Alpine Merino sheep by using weaning body weight (WW) as the target trait. The research integrated genome selection with GWAS analysis and compared it to the best linear unbiased prediction method for the genome. The findings are intended to offer technical support for future Alpine Merino sheep breeding and improve the accuracy of genomic breeding value estimation.

## 2. Materials and Methods

### 2.1. Materials

The experimental animals in this study were chosen from the Gansu Sheep Breeding Technology Promotion Station in Huangcheng Town, Sunan Yugu Autonomous County, Zhangye City, Gansu Province. The station maintains a strictly standardized breeding and management system to ensure that all animals are fed and cared for consistently. A total of 1007 Alpine Merino ewes were included in the study, with their body weight measured at three months of age. To minimize experimental error, the animals underwent a 12 h fasting period under natural conditions before being weighed on a calibrated automatic scale. Weight measurements were recorded to two decimal places in kilograms. The population was then randomly divided into two groups using the RAND function, with each group serving as the other’s control. One group was designated as the discovery population for GWAS analysis, from which the top 5%, top 10%, top 15%, and top 20% most significant SNPs were selected as a priori marker information. The other group was then merged with the prior marker information from various sets to calculate genetic parameters and evaluate the prediction accuracy of genome breeding value. The accuracy was assessed using a five-fold cross-validation method, and the two groups were cross-validated to compare the prediction accuracy of the two strategies.

At 3 months of age, each sheep had 3 mL blood samples collected via the jugular vein method and stored in a vacuum sampling container with ethylenediaminetetraacetic acid (EDTA) for genotyping. Genotyping was performed using the fine wool sheep 50 K liquid chip (MolBreeding Biotech Ltd., Shijiazhuang, China). The reference genome used was the Oar v4.0 (GCF 000298735.2) version published on NCBI. After quality control and filtering using PLINK software (version: v1.9b4) and Beagle software (version 5.0), a total of 1007 individuals and 41,956 SNPs were retained for subsequent correlation analysis. The study applied filters such as --geno, --mind, --maf, and --hwe to enhance the quality of each SNP locus. The specific quality control process involved eliminating 534 SNP loci based on locus detection rate, retaining all individuals based on genotype deletion rate, removing 285 SNP loci based on the minor allele frequency threshold, and excluding 3434 SNP loci based on Hardy–Weinberg equilibrium conditions.

### 2.2. GWAS Model Analysis Methods

Two experimental groups were utilized in this study: an a priori information discovery group and an a priori information verification group. GWAS analysis was conducted using the a priori information discovery population. The FarmCPU model within rMVP software (version 1.0.7) was employed for GWAS analysis of WW. This model offers distinct advantages in addressing population structure and genetic relationships in GWAS, combining fixed effects and random effects to enhance analysis. The fixed effect accounts for population structure and other confounding factors, and the random effect considers genetic correlations within the population, reducing false positives and improving true association detection [21]. Previous research has demonstrated a significant impact of flock and birth type on weaning weight. Therefore, this study incorporated these factors as fixed effects. The FarmCPU model is outlined as follows:

(1) $$y_{i}=M_{i1}b_{1}+M_{i2}b_{2}+\cdots +M_{in}b_{n}+Z_{ij}u_{j}+e_{i},$$

(2) $$y_{i}=V_{i}+e_{i},$$

In Equations (1) and (2), Equation (1) represents a fixed effect model, and Equation (2) represents a random effect model. Here, $y_{i}$ denotes the phenotypic observation value of the i individual, $M_{in}$ signifies the classification result of n potential related loci in the model, $b_{n}$ is the corresponding effect value to the locus, $Z_{ij}$ represents the classification result of the j marker of the i individual, $u_{j}$ is the effect value of $Z_{ij}$, $V_{i}$ stands for the total genetic effect of the i individual, and $e_{i}$ is the residual vector following a normal distribution of e~N(0, $\sigma_{e}^{2}$). Formulas (1) and (2) are used interchangeably in the execution of the FarmCPU model. Each SNP is considered as a fixed factor for regression analysis, and a significance test is conducted to determine the *p* value of each SNP. The top 5%, top 10%, top 15%, and top 20% SNPs with smaller *p* values are then selected, and these SNPs are further weighted based on their heritability to create another set of prior marker information.

### 2.3. GS Model Analysis Method

In this study, GS analysis was conducted using the GBLUP model in ASReml software (version 4.1.0.176) [22]. The GBLUP model, a method for genome selection analysis, is based on a direct approach. It considers the genetic relatedness between individuals by estimating the genetic variance–covariance matrix (also known as the genome relationship matrix) to predict the estimated breeding value of the individual [23]. The prior label information obtained in this study is validated using a prior information verification group. The GBLUP model utilized in this study is outlined as follows:

(3) y=Xb+Zu+e,

In Formula (3), *y* represents the phenotypic value vector of the individual, and b represents the fixed effect vector. The model includes birth type and herd as fixed factors for analysis. u represents the random effect, following a normal distribution with a mean of 0 and variance of ${G\sigma }_{a}^{2}$, denoted as u~*N*(0, ${G\sigma }_{a}^{2}$), where $\sigma_{e}^{2}$ is the genetic variance; G is the genetic relationship matrix between individuals; X and Z are the incidence matrices of b and u; and e represents the random residual vector, following a normal distribution e∼*N*(0, ${I\sigma }_{e}^{2}$). The kinship matrix utilized in GBLUP was proposed by VanRaden [22].

(4) $$G=\frac{MM^{\prime }}{2\sum_{i=1}^{m}p_{i}(1-p_{i})},$$

In Equation (4), M represents the m×n standardized genotype matrix, where m is the number of markers, and n is the number of genotyped individuals, with $p_{i}$ being the minimum allele frequency of the i locus.

The method for constructing the genetic relationship matrix in GS, combined with GWAS prior information, involves creating two matrices: *G*_1_, using significant prior marker information from GWAS results, and *G*_2_, using the remaining SNP sites. The genetic variance of the WW trait in *G*_1_ and *G*_2_ is then calculated, and a new matrix *G*_3_ is formed by weighting the proportion of explained genetic variance. The equation of *G*_3_ is as follows:

(5) $$G_{3}={\gamma G}_{1}+(1-\gamma )G_{2},$$

In Formula (5), $\gamma =\frac{\sigma_{G1}^{2}}{\sigma_{G1}^{2}+\sigma_{G2}^{2}}$, with $\sigma_{G1}^{2}$ and $\sigma_{G_{2}}^{2}$ representing the genetic variances of *G*_1_ and *G*_2_ matrices, respectively.

### 2.4. Methods for Evaluating Predictive Accuracy of GS

The current genome prediction selection accuracy relies on calculating the Pearson correlation coefficient between GEBV and TBV, a method introduced by Meuwissen in 2001 [4]. This study utilized a five-fold cross-validation approach to assess the accuracy of genomic breeding values [24]. The GS analysis population was randomly divided into 5 groups, with 4 combined as the reference group and the remaining one used for validation. The prediction accuracy was determined by dividing the Pearson correlation coefficient between the obtained genomic breeding value and the phenotypic value by the square of heritability. The average value served as the benchmark for evaluating prediction accuracy. The calculation formula is detailed as follows:

(6) $$R_{\left({y,y}_{pre}\right)}=\frac{\sum_{i=1}^{N}\left(y_{i}-\bar{y})(y_{i,pre}-\bar{y_{pre}}\right)}{\sqrt{\sum_{i=1}^{N}{\left(y_{i}-\bar{y}\right)}^{2}}\sqrt{\sum_{i=1}^{N}{\left(y_{i,pre}-\bar{y_{pre}}\right)}^{2}}},$$

In Formula (6), y and $y_{pre}$ represent the predicted value (GEBV) and observed value (TBV), respectively.

## 3. Results

### 3.1. Statistics and Distribution of Phenotypic Data

The WW of 1007 Alpine Merino sheep was meticulously documented. To assess the precision of the GBLUP model versus genomic prediction with GWAS information, the phenotype data of the 1007 ewes were randomly split into two groups using the RAND function. Group 1 consisted of 503 ewes, and group 2 consisted of 504 ewes. Each group served as the control group for comparative analysis. The results of descriptive statistical analysis can be found in Table 1.

### 3.2. Genotype Data

**Figure 1:**
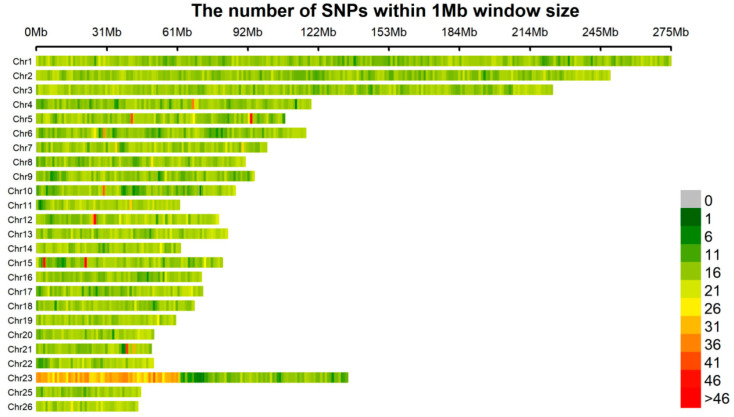
Chromosome-specific SNP density in 1 Mb genomic intervals. The number of SNPs is represented in a green to red scale.

A total of 41,956 SNPs suitable for GWAS and GS were derived through quality control and genotype imputation of data from the 50 K Illumina chip for Alpine Merino sheep. Figure 1 displays the map of marker densities across 26 autosomes.

**Figure 2:**
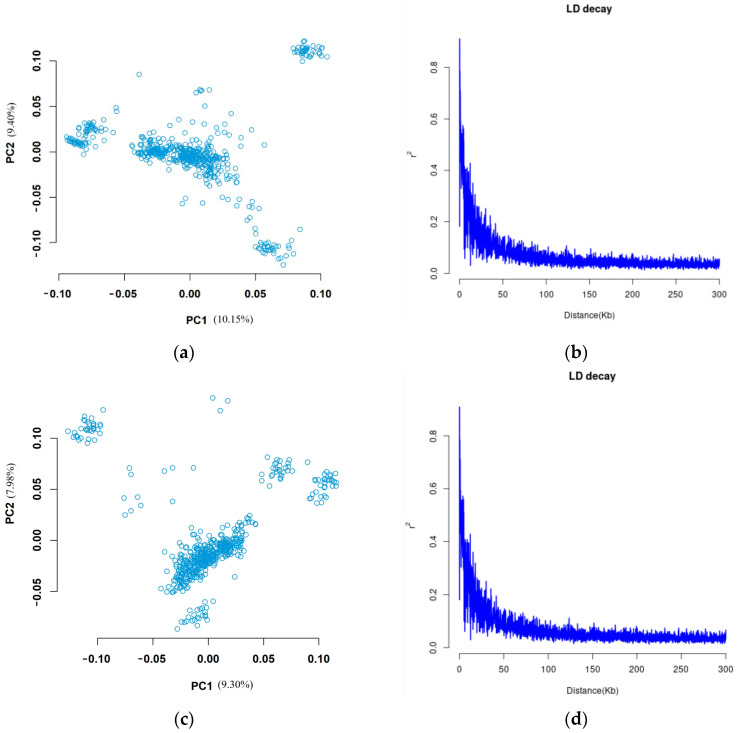
Analysis of group 1 and group 2 population structure and attenuation of LD in the Atlas. (**a**) Group 1 PCA diagram; (**b**) group 1 LD attenuation diagram; (**c**) group 2 PCA diagram; (**d**) group 2 LD attenuation diagram.

The population structure analysis of group 1 and group 2 was conducted separately, with the results displayed in Figure 2a,c, revealing distinct stratification within the two experimental groups. Additionally, linkage disequilibrium analysis was performed on Alpine Merino sheep, with the results presented in Figure 2b,d.

### 3.3. GWAS Analysis Results

**Figure 3:**
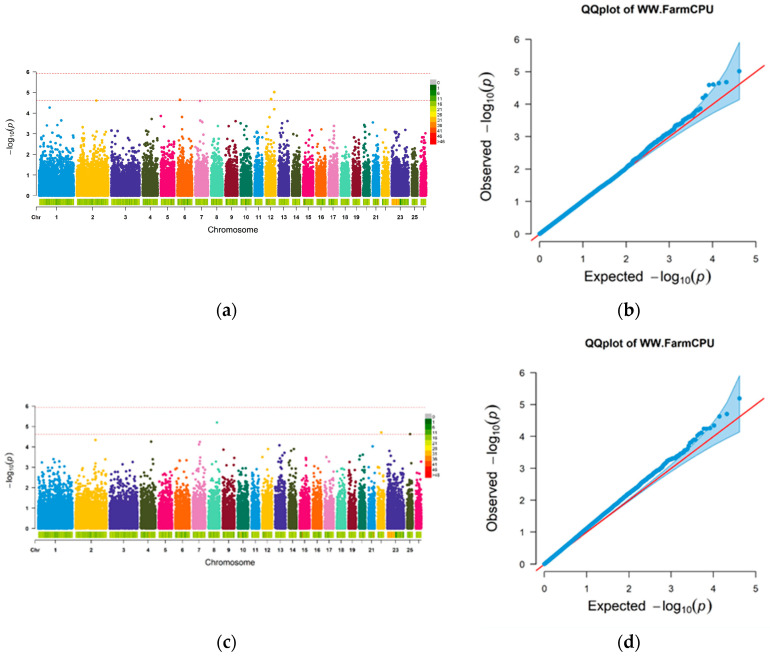
GWAS analysis results. (**a**) Group 1 Manhattan plot; (**b**) group 1 QQ plot; (**c**) group 2 Manhattan plot; (**d**) group 2 QQ chart.

Utilizing the FarmCPU model, this study examined the relationship between group 1 and group 2 based on measured WW traits. The results, including a Manhattan plot and QQ plot, can be seen in Figure 3. Additionally, a subset of the most significant SNPs, comprising the top 5%, top 10%, top 15%, and top 20%, was identified as a set of prior marker information. Specifically, the top 5% yielded 2000 SNPs, and the top 10% had 4000 SNPs. The top 15% and top 20% contained 6000 and 8000 SNPs, respectively. The remaining SNPs were distributed across the chromosomes.

### 3.4. GS Analysis Results

Despite the questionable hypothesis underlying the GBLUP model, it still serves as an effective method for selection prediction based on genotype data. In the absence of GWAS results, Table 2 presents the genetic parameters of group 1, with the G matrix yielding a genetic variance of 1.135, an environmental variance of 8.153, and a heritability estimate of 0.122. The prediction accuracy from five-fold cross-validation was determined to be 0.075.

With GWAS results based on group 2, the top 5% most significant SNPs from GWAS were utilized to construct the genetic relationship matrix G_1_, resulting in a genetic variance of 1.022, an environmental variance of 8.275, and a heritability of 0.110. The remaining 95% of SNPs were employed to create G_2_, which showed a genetic variance of 1.124, an environmental variance of 8.166, and a heritability of 0.121. The weighted values for G_1_ and G_2_ were calculated as 0.476 and 0.533, respectively. By combining GWAS prior marker information to form the genetic relationship matrix G_3_, the genetic variance was determined to be 1.161, the environmental variance to be 8.121, and the heritability to be 0.125. The prediction accuracy was 0.073, resulting in a decrease of 2.67% in accuracy.

The study focused on the top 10% of SNPs to create the genetic relationship matrix G_1_, which revealed a genetic variance of 1.369, an environmental variance of 7.930, and a heritability of 0.147. The remaining 90% of SNPs were used to form the genetic relationship matrix G_2_, showing a genetic variance of 1.083, an environmental variance of 8.207, and a heritability of 0.117. The weighted values for G_1_ and G_2_ were determined as 0.558 and 0.442, respectively. Additionally, the genetic variance and environmental variance were calculated as 1.319 and 7.962, respectively, using the genetic relationship matrix G_3_ constructed with prior marker information from GWAS. The heritability was found to be 0.142, with a prediction accuracy of 0.090, representing a 20.00% improvement in accuracy.

The genetic parameters of group 2 are presented in Table 3. Using the G matrix, the genetic variance was calculated to be 3.932, and the environmental variance 6.045, resulting in a heritability of 0.394. The prediction accuracy from a five-fold cross-validation was 0.228. Based on the GWAS analysis of group 1, the top 5% most significant SNPs were selected to form the genetic relationship matrix G_1_. The genetic and environmental variances for G_1_ were 2.853 and 7.063, respectively, with a heritability of 0.288. The remaining 95% of SNPs were used to create the genetic relationship matrix G_2_, which explained a genetic variance of 3.849 and an environmental variance of 6.131, resulting in a heritability of 0.386. The weights assigned to G_1_ and G_2_ were 0.427 and 0.573, respectively. By combining GWAS marker information, the genetic relationship matrix G_3_ was formed, with a genetic variance of 3.922 and an environmental variance of 6.011. The heritability for G_3_ was 0.395, and the prediction accuracy improved to 0.236, representing a 3.51% increase in accuracy.

**Figure 4:**
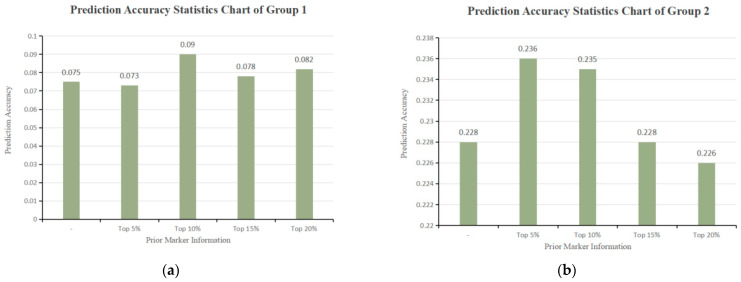
(**a**) Prediction accuracy statistics chart of group 1; (**b**) prediction accuracy statistics chart of group 2.

The study selected the top 20% of SNPs to construct the genetic relationship matrix G_1_, resulting in a calculated genetic variance of 3.430 and an environmental variance of 6.506, with a heritability of 0.345. The remaining 80% of SNPs were used to create matrix G_2_, which explained a genetic variance of 3.849 and an environmental variance of 6.135, yielding a heritability of 0.386. The weights for G_1_ and G_2_ were determined to be 0.472 and 0.528, respectively. Additionally, using the genetic relationship matrix G_3_, which combined GWAS prior marker information, the study found a genetic variance of 3.899 and an environmental variance of 6.036, resulting in a heritability of 0.392 and a prediction accuracy of 0.226. However, the accuracy decreased by 0.88%. The statistical figure of prediction accuracy is shown in Figure 4.

## 4. Discussion

In addition to being influenced by minor polygenes, the weight trait is also impacted by major genes that can have a more significant effect [25]. Currently, the GWAS has become a crucial method for identifying candidate loci related to economic traits in livestock and poultry. Population stratification is a known factor that can influence GWAS results, as it may lead to false positives [26]. PCA is commonly utilized in population-level studies [27]. The PCA analysis of group 1 and group 2 revealed stratification between them. Therefore, the mixed linear model is employed to enhance data accuracy and reduce errors. Various software packages and models are available for GWAS, including the widely used GLM, MLM, FarmCPU model, and others. In this study, the FarmCPU model from the rMVP package was utilized for analysis. FarmCPU, an extension of the compressed MLM, integrates fixed effects and random effects in GWAS analysis [28]. The fixed effect accounts for population structure and other confounding factors, and the random effect considers genetic correlations within the population, reducing false positives and improving true association detection [21]. By combining the strengths of MLM and GLM and addressing their limitations through iteration, FarmCPU can effectively handle highly polygenic traits with numerous genetic variations influencing the phenotype [29]. Overall, utilizing the FarmCPU statistical model for GWAS analysis of WW traits can enhance the accuracy and efficiency in identifying genetic associations by integrating fixed effects and random effects.

GS studies that incorporate prior information face the challenge of effectively utilizing this information. In this study, the GBLUP model was chosen for GS analysis. Though assuming all SNPs have the same effect and distribution is unrealistic in the GBLUP model, it has the advantage of accommodating multiple G matrices and allowing for their weighting [30]. Various researchers have enhanced the GS model by integrating prior information, with advancements such as genomic features BLUP [31] and BayesRC [32]. For instance, Zhang et al. [33] introduced TABLUP in 2010, which optimizes the genome relationship matrix and constructs a trait-specific relationship matrix to improve prediction accuracy. On the other hand, the BayesB-based method is computationally intensive and complex, offering limited benefits to GBLUP accuracy. In 2015, a method based on rrBLUP was proposed, which calculates the effect of each marker, sorts them based on their magnitude, and constructs two matrices for weighted addition, resulting in a significant enhancement in prediction accuracy [34,35].

In this study, a genome-wide significance level of 1 (1/Nsnp) was utilized, and the Bonferroni correction method was applied for multiple tests to identify significant loci. The GWAS analysis of weaning weight traits demonstrated a well-fitted Q-Q diagram, suggesting the model’s validity and the reliability of the results. A total of five significantly associated SNPs were identified, with candidate genes linked to body weight traits found on chromosomes 6, 8, 12, etc. These findings align with previous studies by Yuan et al. [36] and Liu et al. [37], which highlighted the role of *FAM184B*, *NCAPG*, and *LCORL* genes on chromosome 6 in sheep growth, carcass traits, and meat quality. SNP loci were sorted based on the *p* value from GWAS analysis, ranging from the top 5% to top 20% most significant SNPs, which were then integrated into the GBLUP model as prior information. Cross-validation was employed to predict the GS of the second group using the GWAS results of the first group, and vice versa. The prediction accuracy of the GBLUP model-based GS method in both sample groups ranged from 0.075 to 0.228. This was not a significant difference compared to previous studies on pig carcass weight [38], sheep carcass traits [39], and Hanwoo beef cattle weight [40]. When constructing the genome correlation matrix (G matrix) using GWAS results as prior information, the prediction accuracy ranged from 0.073 to 0.236. Notably, utilizing GWAS results from the second group as prior information for predicting the first group led to varying SNP selections based on different GWAS result percentiles, impacting prediction accuracy differently. Overall, increasing the number of prior markers initially increased genetic variance, heritability explained by prior marker information, and GS prediction accuracy before reaching a peak and then declining. These findings align with previous studies by Li [35], Schrag [41], and Gao [42], suggesting that incorporating GWAS prior information enhances GS prediction accuracy. The limited number of prior information sites identified using 50 K liquid chip data may necessitate more accurate screening of prior information through future data resequencing or due to the small sample size. Despite other potential interference factors, the G matrix created from GWAS prior information can enhance the prediction accuracy of GS compared to traditional breeding methods, thereby positively impacting the overall accuracy of genome selection.

Although GS has some limitations, its accuracy can be enhanced by utilizing GWAS analysis results to construct the G matrix. This improvement positively impacts the precision of genome selection, which is widely utilized in practical production. Ongoing model enhancements are being made [43], considering that different populations have distinct breeding objectives and genetic backgrounds. Therefore, when applying genome selection in production, it is crucial to choose prior information and a GS model suitable for the population biology, adjusting relevant parameters as needed. Future studies should explore other methods that combine GWAS and genome selection to further optimize prediction accuracy for handling larger-scale data analysis.

## 5. Conclusions

This study focused on Alpine Merino ewes, utilizing the top 5% to 20% SNPs with small *p* values from GWAS analysis as prior information. By integrating this data with the GS model, the research analyzed genetic parameter estimation and prediction accuracy of weaning weight traits. Incorporating the top 5% to top 20% significant SNPs from GWAS results as prior information into GS can improve the accuracy of predicting genomic breeding value. This improvement in accuracy can offer valuable support for more precise prediction of the genomic breeding value of alpine Merino sheep.

## Figures and Tables

**Table 1:** Descriptive statistics of WW.

Item	Number	Mean	Sd	Median	Trimmed	Med	Min	Max	Se
All	1007	28.27	3.19	28.00	28.21	2.97	18.00	37.60	0.10
Group 1	503	28.12	3.18	28.00	28.07	2.97	18.00	37.60	0.14
Group 2	504	28.42	3.19	28.20	28.34	3.26	19.00	37.00	0.14

Sd stands for standard deviation, Med indicates the median, Min indicates the minimum, and Max indicates the maximum.

**Table 2:** Genetic parameters of group 1.

Prior Marker Information	Matrix	Genetic Variance	Environmental Variance	Heritability	Weight	Prediction Accuracy	Promotion
-	G	1.135	8.153	0.122	-	0.075	-
Top 5%	G_1_	1.022	8.275	0.110	0.476	-	-
	G_2_	1.124	8.166	0.121	0.533	-	-
	G_3_	1.161	8.121	0.125	-	0.073	-2.67%
Top 10%	G_1_	1.369	7.930	0.147	0.558	-	-
	G_2_	1.083	8.207	0.117	0.442	-	-
	G_3_	1.319	7.962	0.142	-	0.090	+20.00%
Top 15%	G_1_	1.085	8.207	0.117	0.492	-	-
	G_2_	1.121	8.169	0.121	0.508	-	-
	G_3_	1.143	8.136	0.123	-	0.078	+4.00%
Top 20%	G_1_	1.282	8.009	0.138	0.546	-	-
	G_2_	1.068	8.223	0.115	0.454	-	-
	G_3_	1.228	8.050	0.132	-	0.082	+9.33%

**Table 3:** Genetic parameters of group 2.

Prior Marker Information	Matrix	Genetic Variance	Environmental Variance	Heritability	Weight	Prediction Accuracy	Promotion
-	G	3.932	6.045	0.394	-	0.228	-
Top 5%	G_1_	2.853	7.063	0.288	0.427	-	-
	G_2_	3.849	6.131	0.386	0.573	-	-
	G_3_	3.922	6.011	0.395	-	0.236	+3.51%
Top 10%	G_1_	3.337	6.590	0.336	0.469	-	-
	G_2_	3.789	6.187	0.380	0.531	-	-
	G_3_	4.049	5.894	0.407	-	0.235	+3.07%
Top 15%	G_1_	3.406	6.529	0.343	0.472	-	-
	G_2_	3.824	6.156	0.383	0.528	-	-
	G_3_	3.937	6.000	0.396	-	0.228	0
Top 20%	G_1_	3.430	6.506	0.345	0.472	-	-
	G_2_	3.849	6.135	0.386	0.528	-	-
	G_3_	3.899	6.036	0.392	-	0.226	−0.88%

## Data Availability

Data will be available upon request from the corresponding author.

## References

[1] Gebreselassie G., Berihulay H., Jiang L., Ma Y. (2019). Review on genomic regions and candidate genes associated with economically important production and reproduction traits in sheep (*Ovies aries*). Animals.

[2] Meuwissen T., Hayes B., Goddard M. (2016). Genomic selection: A paradigm shift in animal breeding. Anim. Front..

[3] Heffner E.L., Sorrells M.E., Jannink J.L. (2009). Genomic selection for crop improvement. Crop Sci..

[4] Meuwissen T.H., Hayes B.J., Goddard M.E. (2001). Prediction of total genetic value using genome-wide dense marker maps. Genetics.

[5] Hayes B.J., Visscher P.M., Goddard M.E. (2009). Increased accuracy of artificial selection by using the realized relationship matrix. Genet. Res..

[6] Habier D., Tetens J., Seefried F.-R., Lichtner P., Thaller G. (2010). The impact of genetic relationship information on genomic breeding values in German Holstein cattle. Genet. Sel. Evol..

[7] Schaeffer L.R. (2006). Strategy for applying genome-wide selection in dairy cattle. J. Anim. Breed. Genet..

[8] Goddard M.E., Hayes B.J. (2007). Genomic selection. J. Anim. Breed. Genet..

[9] Yin L.L., Ma Y.L., Xiang T., Zhu M.J., Yu M., Li X.Y., Liu X.L., Zhao S.H. (2019). The progress and prospect of genomic selection models. Acta Vet. Zootech. Sin..

[10] Esfandyari H., Sørensen A.C., Bijma P. (2015). A crossbred reference population can improve the response to genomic selection for crossbred performance. Genet. Sel. Evol..

[11] Song H., Ye S., Jiang Y., Zhang Z., Zhang Q., Ding X. (2019). Using imputation-based whole-genome sequencing data to improve the accuracy of genomic prediction for combined populations in pigs. Genet. Sel. Evol..

[12] Lourenco D.A., Tsuruta S., Fragomeni B.O., Masuda Y., Aguilar I., Legarra A., Bertrand J.K., Amen T.S., Wang L., Moser D.W. (2015). Genetic evaluation using single-step genomic best linear unbiased predictor in American Angus. J. Anim. Sci..

[13] Zhao Z.D., Zhang L. (2019). Applications of genome selection in sheep breeding. Yi Chuan.

[14] Wolc A., Zhao H.H., Arango J., Settar P., Fulton J.E., O’sullivan N.P., Preisinger R., Stricker C., Habier D., Fernando R.L. (2015). Response and inbreeding from a genomic selection experiment in layer chickens. Genet. Sel. Evol..

[15] Goddard M.E., Kemper K.E., MacLeod I.M., Chamberlain A.J., Hayes B.J. (2016). Genetics of complex traits: Prediction of phenotype, identification of causal polymorphisms and genetic architecture. Proc. Biol. Sci..

[16] Yang J., Zeng J., Goddard M.E., Wray N.R., Visscher P.M. (2017). Concepts, estimation and interpretation of SNP-based heritability. Nat. Genet..

[17] Lopes M.S., Bovenhuis H., van Son M., Nordbø Ø., Grindflek E.H., Knol E.F., Bastiaansen J.W. (2017). Using markers with large effect in genetic and genomic predictions. J. Anim. Sci..

[18] Zhang Z., Ober U., Erbe M., Zhang H., Gao N., He J., Li J., Simianer H. (2014). Improving the accuracy of whole genome prediction for complex traits using the results of genome wide association studies. PLoS ONE.

[19] Yurchenko A.A., Deniskova T.E., Yudin N.S., Dotsev A.V., Khamiruev T.N., Selionova M.I., Egorov S.V., Reyer H., Wimmers K., Brem G. (2019). High-density genotyping reveals signatures of selection related to acclimation and economically important traits in 15 local sheep breeds from Russia. BMC Genom..

[20] Ghasemi M., Zamani P., Vatankhah M., Abdoli R. (2019). Genome-wide association study of birth weight in sheep. Animal.

[21] Liu X., Huang M., Fan B., Buckler E.S., Zhang Z. (2016). Iterative Usage of Fixed and Random Effect Models for Powerful and Efficient Genome-Wide Association Studies. PLoS Genet..

[22] VanRaden P.M. (2008). Efficient methods to compute genomic predictions. J. Dairy. Sci..

[23] Zhang J., Liu F., Reif J.C., Jiang Y. (2021). On the use of GBLUP and its extension for GWAS with additive and epistatic effects. G3 Genes Genomes Genet..

[24] Browne N. (2000). Cross-Validation Methods. J. Math. Psychol..

[25] Mehrban H., Lee D.H., Moradi M.H., IlCho C., Naserkheil M., Ibáñez-Escriche N. (2017). Predictive performance of genomic selection methods for carcass traits in Hanwoo beef cattle: Impacts of the genetic architecture. Genet. Sel. Evol..

[26] Tian C., Gregersen P.K., Seldin M.F. (2008). Accounting for ancestry: Population substructure and genome-wide association studies. Hum. Mol. Genet..

[27] Ma S., Shi G. (2020). On rare variants in principal component analysis of population stratification. BMC Genet..

[28] Alemu A., El Baouchi A., El Hanafi S., Kehel Z., Eddakhir K., Tadesse W. (2021). Genetic analysis of grain protein content and dough quality traits in elite spring bread wheat (*Triticum aestivum*) lines through association study. J. Cereal Sci..

[29] Kusmec A., Schnable P.S. (2018). Farm CPU pp: Efficient large-scale genomewide association studies. Plant Direct..

[30] Ren D., An L., Li B., Qiao L., Liu W. (2020). Efficient weighting methods for genomic best linear-unbiased prediction (BLUP) adapted to the genetic architectures of quantitative traits. Heredity.

[31] Edwards S.M., Sørensen I.F., Sarup P., Mackay T.F., Sørensen P. (2016). Genomic Prediction for Quantitative Traits Is Improved by Mapping Variants to Gene Ontology Categories in Drosophila melanogaster. Genetics.

[32] MacLeod I., Bowman P., Vander Jagt C., Haile-Mariam M., Kemper K., Chamberlain A., Schrooten C., Hayes B.J., Goddard M. (2016). Exploiting biological priors and sequence variants enhances QTL discovery and genomic prediction of complex traits. BMC Genom..

[33] Zhang Z., Liu J., Ding X., Bijma P., de Koning D.-J., Zhang Q. (2010). Best linear unbiased prediction of genomic breeding values using a trait-specific marker-derived relationship matrix. PLoS ONE.

[34] Zhang Z., Erbe M., He J., Ober U., Gao N., Zhang H., Simianer H., Li J. (2015). Accuracy of whole-genome prediction using a genetic architecture-enhanced variance-covariance matrix. G3 Genes Genomes Genet..

[35] Li C., Li J., Wang H., Zhang R., An X., Yuan C., Guo T., Yue Y. (2023). Genomic Selection for Live Weight in the 14th Month in Alpine Merino Sheep Combining GWAS Information. Animals.

[36] Yuan Z., Sunduimijid B., Xiang R., Behrendt R., Knight M.I., Mason B.A., Reich C.M., Prowse-Wilkins C., Vander Jagt C.J., Chamberlain A.J. (2021). Expression quantitative trait loci in sheep liver and muscle contribute to variations in meat traits. Genet. Sel. Evol..

[37] Liu Z., Bai C., Shi L., He Y., Hu M., Sun H., Peng H., Lai W., Jiao S., Zhao Z. (2022). Detection of selection signatures in South African Mutton Merino sheep using whole-genome sequencing data. Anim. Genet..

[38] Iqbal A., Choi T.-J., Kim Y.-S., Lee Y.-M., Zahangir Alam M., Jung J.-H., Choe H.-S., Kim J.-J. (2019). Comparison of genomic predictions for carcass and reproduction traits in Berkshire, Duroc and Yorkshire populations in Korea. Anim. Biosci..

[39] Bolormaa S., Gore K., van der Werf J.H.J., Hayes B.J., Daetwyler H.D. (2015). Design of a low-density SNP chip for the main Australian sheep breeds and its effect on imputation and genomic prediction accuracy. Anim. Genet..

[40] Naserkheil M., Lee D.H., Mehrban H. (2020). Improving the accuracy of genomic evaluation for linear body measurement traits using single-step genomic best linear unbiased prediction in Hanwoo beef cattle. BMC Genet..

[41] Schrag T.A., Westhues M., Schipprack W., Seifert F., Thiemann A., Scholten S., Melchinger A.E. (2018). Beyond Genomic Prediction: Combining Different Types of omics Data Can Improve Prediction of Hybrid Performance in Maize. Genetics.

[42] Gao N., Teng J., Ye S., Yuan X., Huang S., Zhang H., Zhang X., Li J., Zhang Z. (2018). Genomic Prediction of Complex Phenotypes Using Genic Similarity Based Relatedness Matrix. Front. Genet..

[43] Yin L., Zhang H., Zhou X., Yuan X., Zhao S., Li X., Liu X. (2020). KAML: Improving genomic prediction accuracy of complex traits using machine learning determined parameters. Genome Biol..

